# Muscarinic Receptors in Developmental Axonal Competition at the Neuromuscular Junction

**DOI:** 10.1007/s12035-022-03154-1

**Published:** 2022-12-17

**Authors:** Josep Tomàs, Maria A. Lanuza, Manel M. Santafé, Víctor Cilleros-Mañé, Laia Just-Borràs, Marta Balanyà-Segura, Aleksandra Polishchuk, Laura Nadal, Marta Tomàs, Neus Garcia

**Affiliations:** Unitat d’Histologia i Neurobiologia (UHN), Facultat de Medicina i Ciències de La Salut, Universitat Rovira I Virgili, Sant Llorenç 21, 43201 Reus, Spain

**Keywords:** Postnatal synapse elimination, Axonal competition, Acetylcholine release, Muscarinic acetylcholine receptors, Serine kinases, Calcium channels

## Abstract

In recent years, we have studied by immunohistochemistry, intracellular recording, and western blotting the role of the muscarinic acetylcholine receptors (mAChRs; M_1_, M_2_, and M_4_ subtypes) in the mammalian neuromuscular junction (NMJ) during development and in the adult. Here, we evaluate our published data to emphasize the mAChRs’ relevance in developmental synaptic elimination and their crosstalk with other metabotropic receptors, downstream kinases, and voltage-gated calcium channels (VGCCs). The presence of mAChRs in the presynaptic membrane of motor nerve terminals allows an autocrine mechanism in which the secreted acetylcholine influences the cell itself in feedback. mAChR subtypes are coupled to different downstream pathways, so their feedback can move in a broad range between positive and negative. Moreover, mAChRs allow direct activity-dependent interaction through ACh release between the multiple competing axons during development. Additional regulation from pre- and postsynaptic sites (including neurotrophic retrograde control), the agonistic and antagonistic contributions of adenosine receptors (AR; A_1_ and A_2A_), and the tropomyosin-related kinase B receptor (TrkB) cooperate with mAChRs in the axonal competitive interactions which lead to supernumerary synapse elimination that achieves the optimized monoinnervation of musculoskeletal cells. The metabotropic receptor-driven balance between downstream PKA and PKC activities, coupled to developmentally regulated VGCC, explains much of how nerve terminals with different activities finally progress to their withdrawal or strengthening.

## Introduction

During the nervous system development, an overproduction of neurons and synapses generates an extensive connectivity that is corrected by an activity-dependent reduction that refines the specificity of the neuronal circuit [1–4]. The final specificity is attributable to the appropriate matching between the origin and the target of the nerve fibers. Thus, Hebbian competition between nerve endings with different activities leads to the elimination or strengthening of their synapses [5–7]. This developmental synaptic elimination occurs throughout the nervous system, representing a basic mechanism of sinaptogenesis [2, 8–14].

During development, skeletal myocytes start polyinnervated by several axons [15–17], but after their competitive interactions, neuromuscular junctions (NMJ) finally become innervated by only one axon [6, 13, 14, 18]. There are many reviews about synapse elimination mainly focused on the NMJ [1, 18–27]. Several relevant cues of the molecular and cellular mechanisms involved in the elimination of supernumerary nerve terminals have been investigated and collected in the cited reviews. The non-competitive reduction in the number of nerve endings which a motoneuron can support has been studied and characterized. Moreover, it seems also that there is an initial and continuous exchange of the appropriate nerve terminals to produce homogenous fiber-type motor units. At this motorneuron level, [22, 28] show that neuromuscular synapse elimination was accelerated in mutant mice lacking connexin 40, a developmentally regulated gap junction protein, expressed in motor and other spinal neurons, to facilitate electrical coupling.

A major factor, however, is the level of activity of each nerve terminal in a poliinnervated NMJ, and, on the whole, the axonal loss is retarded at low levels of activity and accelerated at increased levels. During the axonal competition, the various nerve endings in a NMJ have a mutual influence on one another and on the postsynaptic muscle cell and the terminal Schwann cell. In adults, terminal Schwann cells sense the release of ACh and ATP from the nerve through M_1_ and A_1_ receptors and in turn influence transmitter release [29, 30]. Terminal Schwann cells have been involved also in axonal competition during development. Glial activity induces synaptic potentiation (through presynaptic adenosine 2_A_ receptors) of strong input in dual junctions but not in weak terminals [31]. Roche et al., [32] using mice lacking Nfasc155, a glial protein detected a delay in postnatal synapse elimination at the NMJ. Moreover, neuregulin-1 signaling between terminal axons and glia during development influences glial cell activation and interposition between the terminal and muscle [23] affecting axon loss. Recently, Jung et al. [33] proposed a model that reproduced synapse elimination showing that synapse elimination is accelerated by increased areas of teloglial cell vacancies. Activity-induced changes in the stability of the muscle cell postsynaptic nAChRs can contribute to reduce the synaptic efficacy of ruled-out nerve terminals [34–36]. Several pre- (calcitonin gene-related peptide, CGRP, [37]) and postsynaptic-derived signals (BDNF, [38, 39]) can also influence supernumerary axonal loss by rewarding or punishing certain nerve terminals. Je et al. [40] using genetic manipulations and pharmacological studies show the involvement of endogenous proBDNF and mBDNF in synapse elimination.

Several presynaptic receptors—mainly muscarinic acetylcholine (ACh) autoreceptors (mAChR), adenosine autoreceptors (AR), and tropomyosin-related kinase B receptor (TrkB)—allow the multiple developing nerve terminals to communicate in the competition that leads to synapse loss in the NMJ [41–43]. This communication can occur directly or with the intermediation of the postsynaptic or Schwann cell components of the tripartite synapse. In particular, mAChRs in the motor terminals seem to permit direct competitive interaction between multiple nerve endings through differential activity-dependent ACh release in the shared narrow developing synaptic cleft. The more active endings may directly punish the less active ones and reward themselves [24, 34, 43–45], and asynchronous activity seems to optimize this interaction to promote synapse elimination [46].

The presence of mAChRs in the motor nerve terminal presynaptic membrane is a clear example of an autocrine mechanism in which a secreted product of a cell can externally influence itself as a feedback modulation both during development and in the adult. Interleukin-2 is another example, being produced by and acting on T lymphocytes themselves (in addition of its paracrine action on target macrophages and other immune cells) [47, 48]. In the case of the cholinergic autoreceptors, the presence of several muscarinic subtype molecules coupled to different downstream pathways can move the autoregulation of the neurosecretion in a broad range between positive and negative effects [49–53]. Because of all these reasons, mAChRs are a relevant component of the complex regulation through pre- and post-synaptic activities of the supernumerary synapse elimination conducting to the optimized monoinnervation of the musculoskeletal system.

We have been working in developmental axonal competition and synapse elimination since the late 1970s (for instance [54, 55]), and the molecular mechanisms are far from being fully elucidated today though many questions have been answered [4, 6, 18]. In recent years, we have studied by immunohistochemistry, intracellular recording electrophysiology, western blotting, subcellular fractionation and co-immunoprecipitation, the involvement of the mAChR subtypes in the mammalian NMJ functionality during development and in the adult [3, 56–61]. In the adult, we characterized how M_1_ and M_2_ mAChRs regulate the PKA subunits (catalytic and regulatory), the PKC (PKCβI and ε isoforms), and their exocytotic targets (Munc18-1, SNAP-25, and MARCKS phosphorylation) showing a co-dependent balance between muscarinic auto receptors which orchestrates transmitter release regulation [62, 63]. We analysed also the involvement of altered metabotropic receptor signaling in amyotrophic lateral sclerosis SOD1-G93A mice [64]. Here, we review previously published data to evaluate the relevance of mAChRs during development in synapse elimination and their crosstalk with other receptors, the downstream kinases, and the targeted voltage-gated calcium channels (VGCC).

Our contribution to the understanding of the synapse elimination process can be summarized in this review: (1) the characterization of the presumably most relevant membrane receptors involved, the mAChRs and adenosine receptor subtypes (both allowing a direct paracrine influence between activity-different neighbor competing nerve endings) and the trophic factor receptor TrKB allowing the retrograde influence through BDNF (2) the characterization of the changes in the downstream PKC/PKA ratio (cPKCβI and nPKCε favor axonal retraction) in the competing nerve terminals as a relevant point of the process, and (3) the specific involvement of several VGCC in determining the transmitter release capacity and the retraction of the nerve endings.

## mAChR in Developing NMJ

### mAChR Subtypes in Transmitter Release During Development

We studied the effect of mAChR subtype modulation—mainly in the rodent *Levator auris longus* muscle (LAL)—by comparing neurotransmission at the newborn stage (P7-P9) versus in the monoinnervated mature NMJs (P30). Axonal elimination almost occurs during the first 2 weeks after birth, although a residual 3% of multiply innervated synapses remains at the end of the first month. The period P7-P9 corresponds to the middle of the axonal loss process and the nerve terminal elimination coincides with the morphological maturation of the postsynaptic component on the NMJ. At birth, nearly all of the NMJs (96%) in the LAL muscle are innervated by more than one axon, and at the end of the first week, the percentage of polyinnervation is reduced to nearly 50%. Newborn P7-P9 neurotransmission study includes developing monoinnervated NMJ and developing polyinnervated ones, mainly dual junctions passing the last process of axonal competition. In the latter, strong and weak nerve terminals can be identified by their evoked endplate potentials (EPP), with the least quantal content originating from the weak terminal in dually-innervated junctions [39, 59, 65–68]. Intracellular recordings using muscarinic agonists and blockers show that some of the mAChR subtypes (M_1_, M_2_, and M_4_) influence ACh release both in developing [58–61] and in adult NMJs [41, 69, 70]. Table 1 shows the list of substances used in the described experiments.

**Table 1:** Substances list. Muscarinic, purinergic, TrkB, PKC, PKA substances, calcium channel modulators, calcium ions modulators, and their targets. Stock solutions were prepared using PBS or DMSO in accordance with the commercial product information after preparing the working solution

Substance list					
Muscarinic substances	Abbreviations	Function	Product information	Stock solution	Working solution
Pirenzepine dihydrochloride (PIR)	PIR	M1 antagonist	1071, Tocris Bio	10 mM	10 µM
Methoctramine	MET	M2 antagonist	M105, Sigma- Aldrich	1 mM	1 µM
Muscarinic toxin 3	MT-3	M1 and M4 antagonist	M-140, Alomone	50 µM	100 nM
Muscarinic toxin 7	MT-7	M1 antagonist	Peptides International	50 µM	100 nM
1,1-dimethyl-4-diphenylacetoxypiperidinium iodide	4-DAMP	M3 antagonist	0482, Tocris Bio	100 mM	1 µM
Tropicamide	TRO	M4 antagonist	0909, Tocris Bio	10 mM	1 µM
Purinergic substances					
8-cyclopentyl-1,3-dipropylxanthine	DPCPX	A_1_ R antagonist	C101, Sigma-Aldrich	50 mM	100 nM
2-(2-furanyl)-7-(2-phenylethyl)-7H-pyrazolo[4,3-e][1, 2, 4] triazolo[1,5-c] pyrimidin-5-amine (SCH-58261)	SCH-58261	A_2A_ R antagonist	2270, Tocris Bio	100 mM	50 nM
TrkB substance					
Recombinant human trkB/Fc Chimera	trkB-Fc	TrkB receptor-related substance	688-TK, R&D Systems	100 µg/ml	5 µg/m
PKC substances					
Chelerytrine	Che	PKCs antagonist	C-400, Alomone	10 mM	1 µM
Calphostin C	CaC	PKCs antagonist	C6303, Sigma-Aldrich	2.5 mM	200 nM
Peptide βIV_5–3_	βIV_5–3_	PKCβI selective antagonist	Mochly Rosen, Standford University	10 mM	10 µM
Peptide εV_1–2_	εV_1–2_	PKCε selective antagonist	539,522, Calbiochem	1 mM	10 µM
Bryostatine-1	BRY	PKCs agonist	2283, Tocris Bio	10 µM	1 nM
Phorbol 12-myristate 13-acetate	PMA	PKCs agonist	P1585, Sigma	10 mM	10 nM
12-deoxyphorbol-13-phenylacetate-20-acetate	dPPA	PKCβI selective activator	BML-PE-182–0001, Enzo	1 mg/mL	0.2 µg/mL
2-((2-Pentylcyclopropyl) methyl) cyclopropaneoctanoic acid	FR236924	PKCε selective activator	3091, Tocris Bio	100 mM	100 nM
PKA substances					
N-[2-(p-Bromocinnamylamino)ethyl]-5-isoquinolinesulfonamide dihydrochloride	H89	PKA antagonist	19–141, Millipore-Merck	5 mM	5 µM
8-Bromoadenosine-3′,5′ cyclic monophosphorothioate	Rp8	RI-PKA selective antagonist	129,735–00-8, Biolog	5 mM	100 µM
Adenosine-3′,5′-cyclic monophosphorothioate	Rp	RII-PKA selective antagonist	A002S, Biolog	5 mM	100 µM
Adenosine 3’,5’-cyclic monophosphorothioate,8-bromo-Sp-isomer	Sp8Br	PKA agonist	116,818 Calbiochem	5 mM	10 µM
Calcium channel modulators					
Nitrendipine (NT)	NT	L-type channel blocker	N144, Sigma-Aldrich	50 mM	1 µM
ω-conotoxin-GVIA	ω-CON	N-type channel blocker	C9915, Calbiochem	1 mM	1 µM
ω-Agatoxin IVA	ω-AGA	P/Q-type channel blocker	STA-500, Alomone	100 nM	100 nM
1,4-Dihydro-2,6-dimethyl-5-nitro-4- (2-trifluoromethylphenyl) pyridine-3-carboxylic acid methyl ester	Bay-K8644	L- type calcium channel agonist	B-350, Alomone	50 mM	5 µM
(2R)-2-[(6-{[(5-Methylthiophen-2-yl) methyl]amino}-9-propyl-9H-purin-2-yl)amino]butan-1-ol	GV-58	CaV2.2 and CaV2.1 Ca^2+^ Channels activator	G-140, Alomone	20 mM	20 µM
Calcium ions modulators					
1,2-Bis(2-aminophenoxy) ethane-N,N,N',N'-tetraacetic acid tetrakis acetoxymethyl ester	BAPTA-AM	Ca ^2+^ chelator	Ab120503, Abcam	10 mM	5 µM

In the adult, the M_1_ receptor increases ACh release because its selective block with pirenzepine [PIR] or muscarinic toxin-7 [MT-7] reduces it. On the contrary, the M_2_ receptor reduces ACh release because its selective inhibition with methoctramine [MET] or AFX-116 increases it [41, 52, 58, 71]. The M_3_ (4-DAMP) and M_4_ (tropicamide [TRO] and muscarinic toxin-3 [MT-3]) blockers do not produce any effect in transmitter release. Thus, in the adult, M_1_ and M_2_ receptors influence ACh secretion in a positive and negative feedback, respectively.

**Fig. 1:**
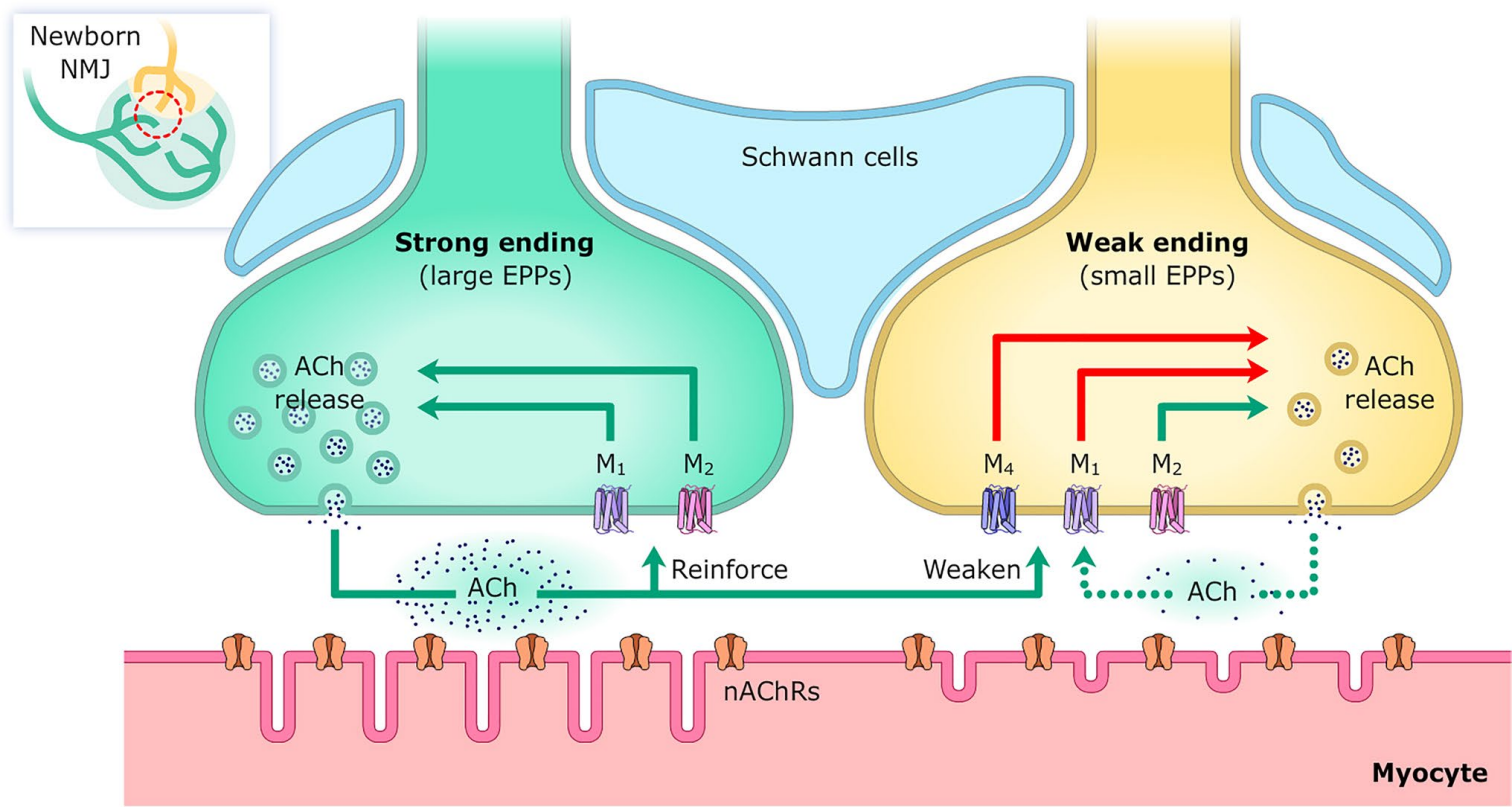
Representation of two neighboring axon terminals in a dually innervated NMJ during development in the common endplate. The strong ending (that evokes the largest EPP) is in green and the weak one (that evokes the smallest EPP) is in yellow. In these nerve endings, muscarinic receptor subtypes and their coupling to ACh release behave differently. In the strong ending (and also in the single endings at the end of the competitive process), both M_1_ and M_2_ enhance ACh release. Using this autocrine mechanism, the strongest endings may reinforce themselves to win the competition. In the weak endings, only M_2_ stimulates the release, whereas M_1_, with the additional involvement of M_4_, reduces the EPP size. Thus, the weakest nerve endings may be negatively influenced by the ACh release from the strongest axons. Inside the nerve terminals, green arrows indicate transmitter release potentiation, and red arrows depression. The effects of M_1_ and M_4_ in weak endings are additive whereas the effects of M_1_ and M_2_ in the strong ones are not. Nicotinic ACh receptors (nAChRs) are represented in the postsynaptic membrane

However, during development, all the selective M_1_ and M_2_ blockers were tested to reduce the release both in the recently monoinnervated NMJ and in the strongest endings of dual junctions (Fig. 1) that are still in the competition at P7-P9. Interestingly, the effect of any M_1_ antagonist was not additive with the effect of any M_2_ antagonist, suggesting the operation of the same mechanism in both cases (i.e., the effect of PIR is completely prevented by a preincubation with MET and *viceversa*). This suggests the commitment of all muscarinic pathways to promote neurotransmission in these nerve endings during maturation. Nevertheless, in the weakest nerve contact in dual junctions, only the M_2_ blockers reduce release whereas M_1_ and M_4_ blockers increase the EPP (Fig. 1) [39, 58–61]. We sequentially added PIR and TRO to the muscle and found that their respective effects on the weak nerve terminals were additive, showing that M_1_- and M_4_-mediated pathways are different in these endings. However, like in the strong and in the monoinnervated junctions, the effect of the M_1_ and M_2_ drugs is still mutually exclusive and not additive. Thus, the two additive M_4_ and M_1_ mechanisms result in a powerful inhibitory modulation that probably overcomes the M_2_ mechanism, which enhances neurotransmission in all kinds of endings during development. Finally, as stated, in the adult monoinnervated NMJs, M_2_ and M_1_ receptors change their coupling to regulate neurotransmission by negative and positive feedback, respectively.

In summary (Fig. 1), an advantage of the more differentiated nerve terminals seems to be the commitment of all mAChR subtypes (M_1_ and M_2_ in this case) to enhance ACh release, and using this autocrine mechanism, the strongest endings may reinforce themselves to win the competition. However, the weakest axon may be negatively influenced by ACh release from the strongest axons through the M_1_ and M_4_ subtype pathways. The described evidence argues in favor of the relevance of local factors explaining, in fact, how an axon that fails at one muscle endplate can win the competition at another [72]. So, a developmentally regulated specific expression of the mAChR subtypes seems to be a relevant mechanism in synaptogenesis.

### mAChR in the Physical Withdrawal of Supernumerary Axons

How the described evidence of the developmental muscarinic modulation of ACh release can be related with supernumerary axon elimination from the NMJ? We favor the hypothesis of the final strengthening and consolidation of the strongest endings in dual junctions because their molecular and functional similarity with the solitary endings in the most mature NMJ. Thus, both M_1_ and M_2_ receptors are coupled to potentiate release in these endings along with the expression in these nerve terminals of a more differentiated VGCC stoichiometry and serine-threonine kinases coupling to ACh release (see later). To investigate this, we made axonal counts in confocal LAL preparations from B6. Cg-Tg (Thy1-YFP)16 Jrs/J mice that express spectral variants of GFP (yellow-YFP) at high levels in motor neurons [43]. Muscles were processed to detect also the postsynaptic nicotinic acetylcholine receptors (nAChRs) with TRITC-α-BTX (Bungarotoxin). We counted the percentage of singly-, dually-, and triply- (or more) innervated synapses at P7, P9, and P15 after 2 (days 5, 6), 4 (days 5–8), and 10 (days 5–14) subcutaneous applications over the LAL muscle surface of the considered muscarinic substance [3, 56, 57].

In P7 mice, we observed that when M_1_ or M_4_ receptors are selectively blocked by PIR or MT3, axonal loss is accelerated (but not when M_2_ is blocked with MET). Thus, at P7 (considering the effect on neurotransmission of the muscarinic receptors, see Fig. 1), M_2_ favors ACh release and possibly the competitive force, related with more transmitter release, in all axons although not affecting the axonal elimination rate. However, M_1_ increases release in the strong axon and decreases it in the weak one (together with M4 in this case), and these tonic effects resulting in a delay in axon loss (evidenced by a conspicuous acceleration when M_1_ or M_4_ receptors are selectively blocked) [43]. We do not know which one can be the prevalence of any of these muscarinic receptors in the different nerve endings at this period, but it is conceivable that mAChR subtypes would participate and even be involved in determining competitive interactions rather than speeding up axonal elimination around P7 [3]. Thus, it appears that at P7, mAChRs-mediated competitive axonal interactions (and also interactions mediated by AR and TrkB receptors—see later) are taking place with the result of an initial delay in synapse elimination because effective axon loss is not yet occurring in most synapses at this moment.

Two days later (P9), the continued exposition to PIR or MET (but not to the M_4_ blocker MT3) for 4 days results in a clear delay in axon loss. This indicates that both M_1_ and M_2_ receptors acquire during this period the role of promoting the full sequence of axonal elimination. Interestingly, MET has a greater ability than PIR to delay the final monoinnervation, indicating the powerful effect of the M_2_ on axon loss at P9, probably potentiating the strongest nerve endings [43]. Joint inhibition of M_2_ and M_1_ (MET + PIR) pathways show that their effect on axonal elimination is not additive, suggesting a shared downstream mechanism at this developmental stage (see below) and the commitment of all muscarinic receptors to promote axon elimination. In the adult, mAChRs show some G protein promiscuity [73, 74] suggesting that M_2_–M_1_ shared developmental mechanism may relate with G protein sharing. The effect on the ACh release of these two receptors may reinforce the increasingly stronger endings and be detrimental to the weak ones. The presence of the M_4_ mAChR subtype in the weakest ending at P7 and their functional disappearance at P9 along with the shift of the M_1_ function (from ACh release reduction in the weakest endings to favoring ACh release in the endings that become stronger) can be important changes in synapse elimination. Interestingly, the progressive change in M_4_ and M_1_ function during NMJ maturation coincides with the slightly later shift of the M_2_ function that will change to negatively modulate ACh release around P15 and for the rest of the adult stage [43]. Therefore, the functional shift of all mAChR types during the maturation of the neuromuscular synapses argues in favor of their relevance in this process. The shift mechanism must be able to explain how M2 changes from positive to negative action on ACh release whereas M_1_ changes reciprocally. The possibility that a change in the expression (protein level) of the M_1_ and M_2_ mAChRs themselves may contribute to explain their functional change needs to be seriously considered. However, the movement of both M_1_ and M_2_ receptors between the extreme positive and negative influences on ACh release during maturation raises some concerns about the possible relevance of the protein level change to *fully* explain these extreme changes in their downstream coupling. Alternatively, as stated, these metabotropic receptors are GPCR, and a developmentally regulated displacement of their coupling between Gs and Gi proteins seems to be an attractive hypothesis. This mechanism may be facilitated because several GPCR function within lipid raft plasma membrane microdomains, which may be important for regulating their signal transduction. In a previous study in the mature NMJ, however [75], we show that the disruption of lipid rafts (methyl-beta-cyclodextrin, 2%) does not change the normal coupling and mutual relations of adenosine receptors and mAChRs on ACh release.

### Relation of the mAChR with AR (A_1_, and A_2A_) and TrkB Receptor on Developmental Axonal Elimination

Even the continued application of M_1_ and M_2_ inhibitors cannot stop axonal loss, which is completed around P15 [43]. This suggests the complex involvement of multiple other pathways including postsynaptic-derived factors [39, 43, 56, 57]. We found that, in addition to the presynaptic mAChR M_1_, M_2_, and M_4_, at least adenosine receptors (AR; A_1_ and A_2A_) and the tropomyosin-related kinase B receptor (TrkB), cooperate in synapse elimination [56, 57]. It seems that this multiple signaling would define the conditioning factors of the axonal competition and thus, the final fate of the individual nerve terminals. However, the achievement of the monoinnervated NMJ may be constitutively regulated.

Concerning to transmitter release, AR is present in the motor terminals of the newborn and adult NMJs [76, 77] and, during development, released adenosine from different components of the synapse may activate both A_1_R and A_2A_R and have a facilitatory action on ACh release. Unfortunately, we do not know the specific involvement of these receptors in the strong and weak nerve endings. Neurotrophins and their receptors are also expressed in both development and adulthood [78–83]. Low doses of BDNF rapidly induce a TrkB-dependent potentiation at developing NMJs in culture [84] and, in ex vivo developing NMJ, BDNF increases ACh release in both the weak and strong endings around P7 [68]).

Concerning to developmental axonal elimination, specific inhibitors reveal that both AR delay axonal loss at P7 but accelerate it at P9. This effect is similar to that of mAChRs. The BDNF-TrkB pathway also plays a biphasic role because BDNF initially delays elimination and subsequently accelerates it at P9 [3]. Thus, several metabotropic receptors overlap and share the common function of modulating a major mechanism of synaptogenesis as can be the definition of the final matching of the synaptic partners. Interestingly, for all receptors, an initial delay in axonal elimination observed at P7 is followed by the acceleration at P9, pointing to the existence of a multifactorial and redundant mechanism aimed at ensuring the specific NMJ monoinnervation. For instance, all receptors (except M_4_) directly accelerate axonal loss at P9. Ranked according to their importance (from more to less), these are M_2_-M_1_-A_1_-A_2A_-TrkB [3].

**Fig. 2:**
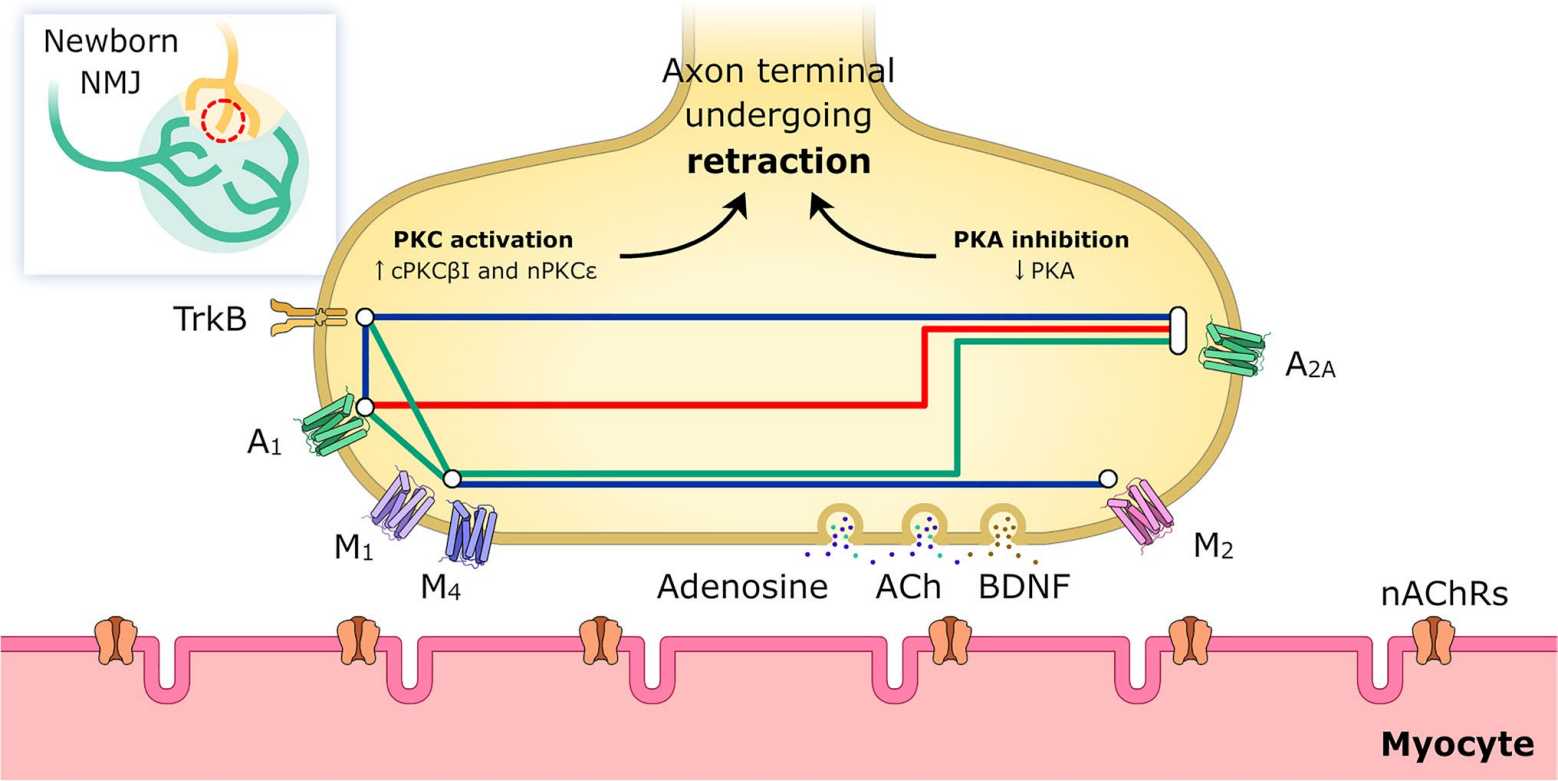
Retracting axon terminal around P9. Metabotropic receptors at the left have downstream pathways linked to PKC activation (M_1_, A_1_, and TrkB; M_4_ is included here because its effect is similar to the M_1_ effect, see the text). The PKC isoforms in the NMJ presynaptic component are cPKCβI and nPKCε. Receptors at the right are linked with PKA inhibition (M_2_ and A_2A_). The individual action of all these six receptors (M_4_ indirectly) promotes or accelerates axonal retraction and loss. Thus, a metabotropic receptor-driven balance between PKA and PKC activities regulates axonal withdrawal. Synergistic or antagonistic crosstalk between mAChR, AR, and TrkB can be revealed by inhibiting two receptors at a time. In the figure, the receptors that are related with a blue link (M_1_/M_4_ with M_2_, A_1_ with TrkB, and TrkB with A_2A_) seem to share the same pathway because their dual inhibition produces the same effect as their individual inhibition over axon loss. On the other hand, M_1_/M_4_ show a synergistic additive behavior (green link) with TrkB, A_1_, and A_2A_. Finally, both AR, A_1_, and A_2A_ show an antagonistic relationship and are mutually occlusive (red link)

Given the observed downstream shared effect of these receptors, we simultaneously applied two selective antagonists to reveal the cooperation between mAChR, AR, and TrkB receptors and the possible additive (synergistic) or occlusive (antagonic) crosstalk between them (Fig. 2) [4, 43, 56, 57]. Studying the adult NMJ, we identified several links between purinergic receptors and mAChRs and found that the functional integrity of mAChRs coupling to the neurotransmission depends on normal purinergic receptors operation. This indicates the clear interaction between both receptor families in the adult [75]. In the newborn, the main results show a synergistic role of the M_1_ mAChR, which potentiates the effect of both AR (A_1_, 58% and A_2A_ 36%) and TrkB (25%) on axonal elimination. On the other hand, though the M_4_ subtype is not directly involved in axonal loss as previously stated, it strongly potentiates the effect of AR (A_1_, 33% and A_2A_ 32%) and TrkB (23%) thus acting similarly as to the M_1_ receptor. Interestingly, a comparable effect of M_1_ and M_4_ is observed on the ACh release capacity of the weakest nerve terminals in dual junctions as shown above (see also Fig. 1). However, as previously stated, M_2_ has the most powerful effect on axon loss and the inhibition of both AR or TrkB receptor does not affect their function. When the TrkB inhibitor TrkB-Fc is associated with one of the AR inhibitors (DPCPX for A_1_, or SCH58261 for A_2A_), the final effect is just the same as the individual effect of one of them on axon loss. When both ARs are blocked simultaneously, occlusion is complete, and the final result is no different from that of the untreated control [57].

Thus, taking these data into consideration, we represented in Fig. 2 the observed relations of the considered receptors that modulate developmental supernumerary axonal loss. In Fig. 2, the receptors that are related with a blue link (M_1_/M_4_ with M_2_, A_1_ with TrkB, and TrkB with A_2A_) seem to share the same pathway because their dual inhibition produces the same effect as their individual inhibition over axon loss. On the other hand, M_1_/M_4_ show a synergistic additive behavior (green link) with TrkB, A_1_, and A_2A_. Finally, both AR, A_1_, and A_2A_ show an antagonistic relationship and are mutually occlusive (red link). All these receptors are involved in promoting supernumerary axonal elimination, and we investigate their downstream links.

## mAChR Coupling to Serine Kinases During Synapse Elimination

The mAChR downstream signaling converges in intracellular effector kinases, mainly the serine-threonine protein kinases A and C (PKA and PKC), which phosphorylate targets involved in synaptic function and axon loss. Receptors and kinases may coordinately regulate the developmental synapse elimination. Generally, M_1_ operates by stimulating PKC whereas M_2_ and M_4_ inhibit PKA. It is known that, in most cells, A_1_, M_1_, and TrkB operate mainly by stimulating the phospholipase C (PLC) and, therefore, PKC and the inositol triphosphate (IP_3_) pathway, whereas A_2A_, M_2_, and M_4_ inhibit the adenyl cyclase (AC) and PKA pathway [50, 51, 85, 86].

At the left of Fig. 2, we represented the receptors coupled to activate PKC (M_1_, A_1_, and TrkB—we added M_4_ due to a connection with M_1_—), and on the right, those that downregulate PKA (M_2_ and A_2A_), and we investigated the hypothesis of the reciprocal involvement of these kinases in synapse elimination. In dually innervated, developing NMJs, the block of PKC (for instance, with calphostin C or chelerytrine) increases ACh release from the weakest nerve terminals (roughly 80%) but does not change ACh release from the strong nerve terminal or even from the more mature monoinnervated junctions. Moreover, after blocking PKC, the mean number of functional axon terminals per synapse increases by about 50%, indicating some recruitment of silent synapses at this time that are probably in the process of disconnection [39, 58–60, 67, 87]. Thus, PKC is involved in reducing neurotransmission in certain weak nerve endings which may facilitate axonal elimination. Recently, we found [88] that PKC favors axon loss through cPKCβI and nPKCε isoform activity (as judging by the effect of their general [Bry-1 or PMA] and specific activators [dPPA, FR236924] and inhibitors [βIV_5–3_ and εV_1–2_] respectively) whereas PKA-I and II activity (as judging by the effect of their specific blockers [H-89, Rp8-Br, and Rp-cAMPs] and activator [Sp8Br], respectively) delay axonal loss in P9 mice. Furthermore, no significant differences exist between the effects of PKA activators and PKC inhibitors, or between PKA inhibitors and PKC activators, on changing axon loss rate [25]. Moreover, a similar level of PKA inhibition and PKC potentiation (mainly of the cPKCβI and nPKCε isoforms that are strictly localized on the presynaptic site [89–91]) seems to be required to advance in axonal loss, clearly suggesting the complementarity of these kinases. On the contrary, the increase of the PKA activity, the reduction of the PKC activity, or, in most cases, both situations simultaneously can reduce synapse elimination [57]. Thus, a metabotropic receptor-driven balance between PKA and PKC activities seems to be involved in synapse elimination and axonal withdrawal as represented in Fig. 2.

It is known that reduction of the postsynaptic activity or contraction results in a delay in synapse elimination during NMJ development [18, 92–95]. In line with this, we made experiments blocking the muscle cell’s contractile activity with μ-conotoxin GIIIB which blocks muscle cell sodium channel but preserves neurotransmission because does not influence the nAChR [26]. Accordingly, incubation with μ-conotoxin GIIIB also results in a delay in axon loss. Thus, a contractile activity-related retrograde influence from the postsynaptic site may contribute to the synapse elimination. The simultaneous application of one presynaptic cPKCβI or nPKCε activator and μ-conotoxin GIIIB fully prevents the postsynaptic contraction block effect on axon loss. Thus, the axonal loss can be altered by acting directly in presynaptic targets (and receptors like mAChRs). Possibly, the above-cited presynaptic PKCs may be modulated by retrograde control (for instance, through BDNF production). This argues in favor of a complex regulation through pre- and post-synaptic activity of the serine-threonine kinases as mediators of the synapse elimination. The regulation of these kinases by mAChR and neurotrophic receptors affecting their phosphorylating activity on targets of the exocytotic vesicular release apparatus (as synapsin I and the SNARE/SM proteins Munc18-1 and SNAP-25) has been described by us in the adult NMJ [96, 97].

## mAChR Coupling to Calcium Channels During Synapse Elimination

### mAChR and Calcium Channels in Transmitter Release During Development

During development, P/Q, N, and α1D-L subtypes of the VGCC are present in the nerve terminals on the LAL muscle. The protein expression of all these channels increases during the development P5-P7 time period. Western blots at P30 show that the P/Q level is at its highest whereas α1D-L and N channel proteins stabilize at a lower level [26].

In dually innervated fibers during NMJ maturation (P7-P9), the block of any of these three VGCC reduces about 2/3 of the EPP produced by the strongest ending [65, 66], indicating the multichannel dependence of calcium entry to promote ACh release in these endings. In the early monoinnervated endplates, the P/Q-type channel blocker ω-Aga-IVA and the N-type blocker ω-CgTx-GVIA still reduce the EPP amplitude (~ 80% and ~ 60%, respectively) whereas the L-type blocker nitrendipine does not anymore. Finally, in the adult (at P30), only the P/Q-type VGCC functionally persists being the only one that, when blocked, strongly inhibits ACh release [69]. However, in the weak ending of dual NMJs, the block of any VGCC channel results in an increase of the size of the evoked EPP, indicating that a part of the calcium entry through all channels can negatively influence transmitter release and even may contribute to disconnect these endings [65, 66].

**Fig. 3:**
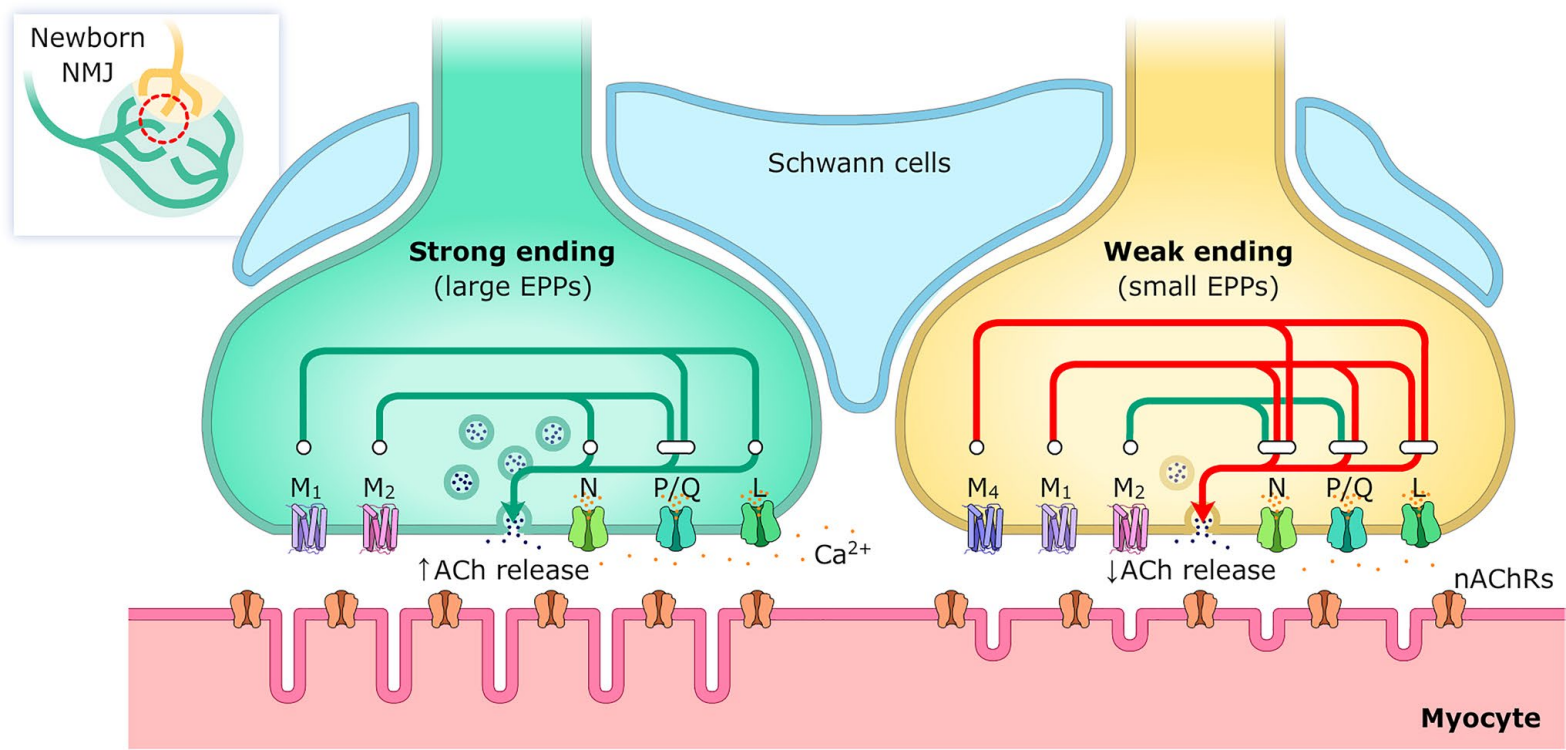
Differential coupling of VGCC and mAChRs in the strong and weak endings in a common endplate. The effect of the mAChR and VGCC on ACh release is shown in green (potentiation) or red (depression). The links between receptors and channels indicate the mutual dependence between these molecules to produce the final effect. In the strongest nerve terminal (shadowed in green), M_1_ receptors need the P/Q- and the L-type VGCC, whereas the M_2_ need the P/Q- and N-type VGCC to potentiate ACh release. The weak nerve ending (in yellow) is represented with the mAChR (M_1_, M_2_, and M_4_) and the VGCC (P/Q, N, and L) subtypes that are operative in these endings. The M_2_ function (also potentiation in these endings), similarly depends on P/Q- and N-type channels, but not on the L-type channel. A leading role for the L channel in axonal loss seems to emerge because of their unique coupling to M_1_ and M_4_ mAChRs, which clearly reduces neurotransmission in the weak nerve terminals presumably in process of elimination. Moreover, the coupling of all muscarinic and metabotropic receptors studied to promote axonal elimination at P9 had a multichannel dependence (P/Q- and L-type) with a relevant role of the L channel

Figure 3 shows that in the strongest endings, there is a differential coupling of the calcium channels with the M_1_ and the M_2_ receptors. M_1_ receptors need the P/Q- and the L-types VGCC whereas the M_2_ effect needs the P/Q- and N-types VGCC. As previously stated, in these strongest ending in dually-innervated synapses, both M_1_ and M_2_ mAChR have an ACh release potentiating effect (see Fig. 1). In the monoinnervated junctions, the ACh release potentiating effect of both M_1_ and M_2_ mAChR relies only on the P/Q-type VDCC because the effect of the receptors is occluded only when this channel is inhibited, even in the presence of high Ca^2+^ concentration [59]. However, in the weak endings, the function of tropicamide-sensitive M_4_ mAChRs did not depend on the P/Q-type VDCCs, although it did depend on the normal function of the L- and N-type channel [39, 58, 59, 61]. In the same weak endings, the pirenzepine-sensitive M_1_ mAChRs function had multichannel dependence (P/Q-, N-, and L-types), and the methoctramine-sensitive M_2_ function also had a multichannel dependence (P/Q- and N-type channels but not the L-type channel). As previously stated, in the weakest nerve contact in dual junctions, only the M_2_ has an ACh release potentiating effect whereas M_1_ and M_4_ reduce release (Fig. 1).

Thus, results indicate that the nerve ending that becomes strong during competition uses a specific coupling (different from the adult) of the M_1_ and M_2_ mAChR with a broad (the three VGCC are involved), well-defined VGCC stoichiometry that favors ACh release. This configuration courses with the maintenance of these axon terminals. On the contrary, it seems that in the weakest nerve ending, the M_2_ release potentiating effect (linked to P/Q and N VGCC) is surpassed by the M_1_/M_4_ effect (linked to the three VGCCs) that depresses ACh release and favors axon elimination.

### mAChR and Calcium Channels in the Withdrawal of Supernumerary Axons

The L and P/Q-type (but not the N-type) channels tonically enhance synapse elimination because their block prolongs the multiinnervation of the developing NMJ, whereas their exogenous stimulation (Bay-K8644 for L channel and GV-58 for P/Q channels) results in a significant acceleration [26]. Moreover, the delaying effect on axon loss of the L and P/Q channels block is equal to that produced by intracellular calcium sequestration with BAPTA-AM. Thus, the calcium entry through these operative channels present in immature nerve endings results in their final loss. The [Ca^2+^]_i_ increase contributes both to transmitter release reduction in certain weak axons (as shown above) as well as nerve terminal loss, and this coincidence argues in favor of a shared mechanism relating transmitter release and axonal competition. It is tempting to speculate about the involvement in the neurite retraction or growth during axonal competition of the differentially expressed Ca(2 +) sensor proteins [NCS-1, Ca(2 +)/calmodulin and several neuro-specific calmodulin-like Ca(2 +) sensor proteins as CaBP1] [98–100]. There is a clear relation between PKA, PKC, and VGCC for developmental axonal loss and synapse elimination. The result after the block of the L-channel (and also after intracellular calcium sequestration) is the same as the inhibition of cPKCβI [25] and from stimulation of PKA [88]. However, the inhibition of the nPKCε produces a greater delay in synapse elimination than the L or P/Q channel block or calcium sequestration. This suggests a VGCC-independent component of the PKC-induced enhancement of axonal withdrawal. In adult NMJ, we have seen that nPKCε promotes the phosphorylation of SNARE/SM proteins Munc18-1 and SNAP-25 in an activity-dependent manner [96, 97]. Similar to the block of L channels, the block of P/Q-type channels produces retardation in axonal loss similar to that observed after cPKCβI inhibition. However, the effect of the P/Q block in delaying axonal loss is smaller than PKA activation, suggesting the relevant involvement of the PKA activity in axonal stabilization [25].

## Concluding Remarks

Axonal competition for synaptic sites is a basic development process that is regulated to achieve optimal connectivity during neurogenesis. In the NMJ, supernumerary axon loss leads to the optimized monoinnervation of the voluntary muscle cells. This process involves activity-dependent autocrine, paracrine (between neighbor nerve terminals), and retrograde (from muscle cells) signalings impacting on the competing nerve terminals. The metabotropic mAChR (M_1_, M_4_, and M_2_ subtypes), purinergic receptors (A_1_ and A_2A_), and TrkB receptors ensure downstream changes in the balance between PKA (favors axonal strengthening) and presynaptic PKC isoforms (cPKCβI and nPKCε favor axonal retraction) activities. mAChRs and kinases pathways differentially couple to P/Q, N, and L subtypes of the VGCC to differently modulate ACh release in the developing nerve terminals (for instance, the strong and weak endings in a dual junction at a given moment during competition). Moreover, calcium inflow through L- and P/Q-type channels could affect nerve terminals depending on their activity, leading to their final withdrawal or strengthening.

Beyond the analyzed competitive interactions between the multiple axons, several uncertainties persist in the understanding of developmental synapse elimination as, for instance, the mechanism of functionality shifting of muscarinic receptors (and other receptors such as adenosine receptors) or the mechanism of calcium-induced retraction of the ruled-out axons along with the molecular mechanism of rewarding strong endings. The described findings contribute to understanding several aspects of the punishment-rewarding interactions between nerve endings and the contribution of postsynaptic retrograde involvement. It can be stated, however, that the inhibition of any one of these pathways only changes the rate of axonal elimination that finally is completed about 2–3 weeks postnatal indicating the complex multifactorial nature of the process. We think that the high number of molecules and different pathways in the cholinergic peripheral NMJ that is directed to the common objective of supernumerary synapse elimination suggests that some of these molecules may contribute to the same function in other neuronal systems. It seems that the multifactorial mechanism works with precision, though an alteration in many possible points may allow malfunctioning of receptors signaling, kinases ratio, or calcium channel balance resulting in the persistence of multiinnervation. In fact, this alteration has been suggested and even has been shown in a number of diseases such as autistic spectrum disorder (ASD) [101–104].

## Data Availability

We believe that our data are not appropriate for the repository databases available in neuroscience.

## Abbreviations

*ACh*: Acetylcholine
*AR*: Adenosine autoreceptors
*αBTX*: α-BungarotoxinBDNF, brain-derived neurotrophic factor
*EPP*: Evoked endplate potentials
*LAL*: Levator auris longus muscle
*nAChRs*: Nicotinic acetylcholine receptors
*mAChR*: Muscarinic acetylcholine receptor
*M*_*1*_: M_1_-type muscarinic acetylcholine receptor
*M*_*2*_: M_2_-type muscarinic acetylcholine receptor
*M*_*4*_: M_4_-type muscarinic acetylcholine receptor
*MET*: Methoctramine
*NMJ*: Neuromuscular junction
*P7*: Postnatal day 7
*P9*: Postnatal day 9
*P15*: Postnatal day 15
*PIR*: Pirenzepine
*PLC*: Phospholipase C
*PKA*: Protein kinase A
*PKC*: Protein kinase C
*TrkB*: Tropomyosin-related kinase B receptor
*TrkB-Fc*: Inhibitor recombinant human TrkB-Fc Chimera
*TRO*: Tropicamide
*VGCC*: Voltage-gated calcium channels

## References

[1] Thompson WJ (1985). Activity and synapse elimination at the neuromuscular junction. Cell Mol Neurobiol.

[2] Bourgeois J, Rakic P (1993). Changes of synaptic density in the primary visual cortex of the macaque monkey from fetal to adult stage. J Neurosci.

[3] Nadal L, Garcia N, Hurtado E (2016). Synergistic action of presynaptic muscarinic acetylcholine receptors and adenosine receptors in developmental axonal competition at the neuromuscular junction. Dev Neurosci.

[4] Tomàs J, Garcia N, Lanuza MA et al (2018) Adenosine receptors in developing and adult mouse neuromuscular junctions and functional links with other metabotropic receptor pathways. Front Pharmacol 9. 10.3389/fphar.2018.00397 10.3389/fphar.2018.00397 PMC5928480 29740322

[5] Fields RD, Nelson PG (1992) A role for glial cells in activity-dependent development of the vertebrate nervous system. pp 133–214 10.1016/s0074-7742(08)60098-7 1587715

[6] Sanes JR, Lichtman JW (1999). Development of the vertebrate neuromuscular junction. Annu Rev Neurosci.

[7] Zorumski CF, Mennerick S (2000). Neural activity and survival in the developing nervous system. Mol Neurobiol.

[8] (1977) Plasticity of ocular dominance columns in monkey striate cortex. Phil Trans R Soc Lond B, Biol Sci 278:377–409. 10.1098/rstb.1977.0050 10.1098/rstb.1977.0050 19791

[9] Huberman AD (2007). Mechanisms of eye-specific visual circuit development. Curr Opin Neurobiol.

[10] Daniel H, Hemart N, Jaillard D, Crepel F (1992) Coactivation of metabotropic glutamate receptors and of voltage-gated calcium channels induces long-term depression in cerebellar Purkinje cells in vitro. Exp Brain Res 90. 10.1007/BF00227245 10.1007/BF00227245 1327859

[11] Hashimoto K, Kano M (2005). Postnatal development and synapse elimination of climbing fiber to Purkinje cell projection in the cerebellum. Neurosci Res.

[12] Lichtman JW (1977). The reorganization of synaptic connexions in the rat submandibular ganglion during post-natal development. J Physiol.

[13] Benoit P, Changeux J-P (1975). Consequences of tenotomy on the evolution of multiineervation in developing rat soleus muscle. Brain Res.

[14] O’Brien RA, Ostberg AJ, Vrbová G (1978). Observations on the elimination of polyneuronal innervation in developing mammalian skeletal muscle. J Physiol.

[15] Redfern PA (1970). Neuromuscular transmission in new-born rats. J Physiol.

[16] Brown MC, Jansen JK, van Essen D (1976). Polyneuronal innervation of skeletal muscle in new-born rats and its elimination during maturation. J Physiol.

[17] Ribchester R, Barry J (1994). Spatial versus consumptive competition at polyneuronally innervated neuromuscular junctions. Exp Physiol.

[18] Jansen J, Fladby T (1990). The perinatal reorganization of the innervation of skeletal muscle in mammals. Prog Neurobiol.

[19] Brown MC, Holland RL, Hopkins WG (1981). Restoration of focal multiple innervation in rat muscles by transmission block during a critical stage of development. J Physiol.

[20] Ridge RMAP (1989). Motor unit organization in developing muscle. Comp Biochem Physiol A Physiol.

[21] Betz WJ, Ribchester RR, Ridge RM (1990). Competitive mechanisms underlying synapse elimination in the lumbrical muscle of the rat. J Neurobiol.

[22] Personius KE, Slusher BS, Udin SB (2016). Neuromuscular NMDA receptors modulate developmental synapse elimination. J Neurosci.

[23] Lee Y (2020). Developmental neuromuscular synapse elimination: activity-dependence and potential downstream effector mechanisms. Neurosci Lett.

[24] Busetto G, Buffelli M, Tognana E (2000). Hebbian mechanisms revealed by electrical stimulation at developing rat neuromuscular junctions. J Neurosci.

[25] Garcia N, Lanuza MA, Tomàs M (2021). PKA and PKC balance in synapse elimination during neuromuscular junction development. Cells.

[26] Garcia N, Hernández P, Lanuza MA (2022). Involvement of the voltage-gated calcium channels L- P/Q- and N-types in synapse elimination during neuromuscular junction development. Mol Neurobiol.

[27] Van Essen D, Newsome W, Bixby J (1982). The pattern of interhemispheric connections and its relationship to extrastriate visual areas in the macaque monkey. J Neurosci.

[28] Personius KE, Chang Q, Mentis GZ (2007). Reduced gap junctional coupling leads to uncorrelated motor neuron firing and precocious neuromuscular synapse elimination. Proc Natl Acad Sci.

[29] Jahromi BS, Robitaille R, Charlton MP (1992). Transmitter release increases intracellular calcium in perisynaptic schwann cells in situ. Neuron.

[30] Robitaille R, Jahromi BS, Charlton MP (1997). Muscarinic Ca ^2+^ responses resistant to muscarinic antagonists at perisynaptic schwann cells of the frog neuromuscular junction. J Physiol.

[31] Darabid H, St-Pierre-See A, Robitaille R (2018). Purinergic-dependent glial regulation of synaptic plasticity of competing terminals and synapse elimination at the neuromuscular junction. Cell Rep.

[32] Roche SL, Sherman DL, Dissanayake K (2014). Loss of glial neurofascin155 delays developmental synapse elimination at the neuromuscular junction. J Neurosci.

[33] Jung JH, Smith I, Mikesh M (2019). Terminal Schwann cell and vacant site mediated synapse elimination at developing neuromuscular junctions. Sci Rep.

[34] Balice-Gordon RJ, Lichtman JW (1994). Long-term synapse loss induced by focal blockade of postsynaptlc receptors. Nature.

[35] Culican SM, Nelson CC, Lichtman JW (1998). Axon withdrawal during synapse elimination at the neuromuscular junction is accompanied by disassembly of the postsynaptic specialization and withdrawal of Schwann cell processes. J Neurosci.

[36] Akaaboune M, Culican SM, Turney SG (1979). Lichtman JW (1999) Rapid and reversible effects of activity on acetylcholine receptor density at the neuromuscular junction in vivo. Science.

[37] Li M-X, Jia M, Jiang H (2001). Opposing actions of protein kinase A and C mediate Hebbian synaptic plasticity. Nat Neurosci.

[38] Garcia N, Tomàs M, Santafe MM (2010). The interaction between tropomyosin-related kinase B receptors and presynaptic muscarinic receptors modulates transmitter release in adult rodent motor nerve terminals. J Neurosci.

[39] Tomàs J, Santafé MM, Lanuza MA (2011). Silent synapses in neuromuscular junction development. J Neurosci Res.

[40] Je HS, Yang F, Ji Y (2013). ProBDNF and mature BDNF as punishment and reward signals for synapse elimination at mouse neuromuscular junctions. J Neurosci.

[41] Santafé MM, Lanuza MA, Garcia N, Tomàs J (2006). Muscarinic autoreceptors modulate transmitter release through protein kinase C and protein kinase A in the rat motor nerve terminal. Eur J Neurosci.

[42] Amaral MD, Pozzo-Miller L (2012). Intracellular Ca ^2+^ stores and Ca ^2+^ influx are both required for BDNF to rapidly increase quantal vesicular transmitter release. Neural Plast.

[43] Nadal L, Garcia N, Hurtado E (2016). Presynaptic muscarinic acetylcholine autoreceptors (M1, M2 and M4 subtypes), adenosine receptors (A1 and A2A) and tropomyosin-related kinase B receptor (TrkB) modulate the developmental synapse elimination process at the neuromuscular junction. Mol Brain.

[44] Sanes JR, Lichtman JW (2001). Induction, assembly, maturation and maintenance of a postsynaptic apparatus. Nat Rev Neurosci.

[45] Personius KE, Balice-Gordon RJ (2001). Loss of correlated motor neuron activity during synaptic competition at developing neuromuscular synapses. Neuron.

[46] Favero M, Busetto G, Cangiano A (2012). Spike timing plays a key role in synapse elimination at the neuromuscular junction. Proc Natl Acad Sci U S A.

[47] Long M, Adler AJ (2006). Cutting edge: paracrine, but not autocrine, IL-2 signaling is sustained during early antiviral CD4 T cell response. J Immunol.

[48] Ross SH, Cantrell DA (2018). Signaling and function of interleukin-2 in T lymphocytes. Annu Rev Immunol.

[49] Caulfield MP (1993). Muscarinic receptors—characterization, coupling and function. Pharmacol Ther.

[50] Felder CC (1995). Muscarinic acetylcholine receptors: signal transduction through multiple effectors. FASEB J.

[51] Caulfield MP, Birdsall NJ (1998). International union of pharmacology. XVII. Classification of muscarinic acetylcholine receptors. Pharmacol Rev.

[52] Slutsky I, Parnas H, Parnas I (1999). Presynaptic effects of muscarine on ACh release at the frog neuromuscular junction. J Physiol.

[53] Nathanson NM (2000). A multiplicity of muscarinic mechanisms: enough signaling pathways to take your breath away. Proc Natl Acad Sci.

[54] Haimann C, Mallart A, Ferré JT, Zilber-Gachelin NF (1981). Interaction between motor axons from two different nerves reinnervating the pectoral muscle of Xenopus laevis. J Physiol.

[55] Haimann C, Mallart A, Ferré JT, Zilber-Gachelin NF (1981). Patterns of motor innervation in the pectoral muscle of adult Xenopus laevis: evidence for possible synaptic remodelling. J Physiol.

[56] Nadal L, Garcia N, Hurtado E et al (2017) Presynaptic muscarinic acetylcholine receptors and TrkB receptor cooperate in the elimination of redundant motor nerve terminals during development. Front Aging Neurosci 9. 10.3389/fnagi.2017.00024 10.3389/fnagi.2017.00024 PMC5296322 28228723

[57] Tomàs J, Garcia N, Lanuza MA et al (2017) Presynaptic membrane receptors modulate ACh release, axonal competition and synapse elimination during neuromuscular junction development. Front Mol Neurosci 10. 10.3389/fnmol.2017.00132 10.3389/fnmol.2017.00132 PMC5432534 28559796

[58] Santafé MM, Salon I, Garcia N (2003). Modulation of ACh release by presynaptic muscarinic autoreceptors in the neuromuscular junction of the newborn and adult rat. Eur J Neurosci.

[59] Santafé MM, Salon I, Garcia N (2004). Muscarinic autoreceptors related with calcium channels in the strong and weak inputs at polyinnervated developing rat neuromuscular junctions. Neuroscience.

[60] Santafé MM, Lanuza MA, Garcia N (2007). Coupling of presynaptic muscarinic autoreceptors to serine kinases in low and high release conditions on the rat motor nerve terminal. Neuroscience.

[61] Santafe MM, Garcia N, Lanuza MA (2009). Presynaptic muscarinic receptors, calcium channels, and protein kinase C modulate the functional disconnection of weak inputs at polyinnervated neonatal neuromuscular synapses. J Neurosci Res.

[62] Cilleros-Mañé V, Just-Borràs L, Tomàs M (2020). The M 2 muscarinic receptor, in association to M 1, regulates the neuromuscular PKA molecular dynamics. FASEB J.

[63] Cilleros-Mañé V, Just-Borràs L, Polishchuk A et al (2021) M _1_ and M _2_ mAChRs activate PDK1 and regulate PKC βI and ε and the exocytotic apparatus at the NMJ. FASEB J 35. 10.1096/fj.202002213R 10.1096/fj.202002213R 34133802

[64] Just-Borràs L, Hurtado E, Cilleros-Mañé V (2019). Overview of impaired BDNF signaling, their coupled downstream serine-threonine kinases and SNARE/SM complex in the neuromuscular junction of the amyotrophic lateral sclerosis model SOD1-G93A mice. Mol Neurobiol.

[65] Santafé MM, Garcia N, Lanuza MA (2001). Calcium channels coupled to neurotransmitter release at dually innervated neuromuscular junctions in the newborn rat. Neuroscience.

[66] Santafé MM, Garcia N, Lanuza MA (2002). Decreased calcium influx into the neonatal rat motor nerve terminals can recruit additional neuromuscular junctions during the synapse elimination period. Neuroscience.

[67] Santafé MM, Garcia N, Lanuza MA (2009). Interaction between protein kinase C and protein kinase A can modulate transmitter release at the rat neuromuscular synapse. J Neurosci Res.

[68] Garcia N, Tomàs M, Santafé MM (2010). The interaction between tropomyosin-related kinase B receptors and presynaptic muscarinic receptors modulates transmitter release in adult rodent motor nerve terminals. J Neurosci.

[69] Santafé MM, Lanuza MA, Garcia N, Tomàs J (2005). Calcium inflow-dependent protein kinase C activity is involved in the modulation of transmitter release in the neuromuscular junction of the adult rat. Synapse.

[70] Santafé MM, Garcia N, Lanuza MA, Tomàs J (2007). Protein kinase C activity affects neurotransmitter release at polyinnervated neuromuscular synapses. J Neurosci Res.

[71] Minic J, Molgó J, Karlsson E, Krejci E (2002). Regulation of acetylcholine release by muscarinic receptors at the mouse neuromuscular junction depends on the activity of acetylcholinesterase. Eur J Neurosci.

[72] Keller-Peck CR, Feng G, Sanes JR (2001). Glial cell line-derived neurotrophic factor administration in postnatal life results in motor unit enlargement and continuous synaptic remodeling at the neuromuscular junction. J Neurosci.

[73] Leurs R, Pena MSR, Bakker RA (2000). Constitutive activity of G protein coupled receptors and drug action. Pharm Acta Helv.

[74] Jakubík J, Janíčková H, Randáková A (2011). Subtype differences in pre-coupling of muscarinic acetylcholine receptors. PLoS ONE.

[75] Santafé MM, Priego M, Obis T (2015). Adenosine receptors and muscarinic receptors cooperate in acetylcholine release modulation in the neuromuscular synapse. Eur J Neurosci.

[76] Garcia N, Priego M, Obis T (2013). Adenosine A1 and A2A receptor-mediated modulation of acetylcholine release in the mice neuromuscular junction. Eur J Neurosci.

[77] Garcia N, Priego M, Hurtado E (2014). Adenosine A2B and A3 receptor location at the mouse neuromuscular junction. J Anat.

[78] Funakoshi H, Belluardo N, Arenas E (1979). (1995) Muscle-derived neurotrophin-4 as an activity-dependent trophic signal for adult motor neurons. Science.

[79] Gonzalez M, Ruggiero FP, Chang Q (1999). Disruption of Trkb-mediated signaling induces disassembly of postsynaptic receptor clusters at neuromuscular junctions. Neuron.

[80] Ip FC, Cheung J, Ip NY (2001). The expression profiles of neurotrophins and their receptors in rat and chicken tissues during development. Neurosci Lett.

[81] Nagano M, Suzuki H (2003). Quantitative analyses of expression of GDNF and neurotrophins during postnatal development in rat skeletal muscles. Neurosci Res.

[82] Pitts EV, Potluri S, Hess DM, Balice-Gordon RJ (2006). Neurotrophin and Trk-mediated signaling in the neuromuscular system. Int Anesthesiol Clin.

[83] Garcia N, Santafé MM, Tomàs M (2010). The glial cell line-derived neurotrophic factor (GDNF) does not acutely change acetylcholine release in developing and adult neuromuscular junction. Neurosci Lett.

[84] Poo M (2001). Neurotrophins as synaptic modulators. Nat Rev Neurosci.

[85] Caulfield MP (1993). Muscarinic receptors–characterization, coupling and function. Pharmacol Ther.

[86] Nathanson NM (2000). A multiplicity of muscarinic mechanisms: enough signaling pathways to take your breath away. Proc Natl Acad Sci U S A.

[87] Santafé MM, Garcia N, Lanuza MA (2009). Presynaptic muscarinic receptors, calcium channels, and protein kinase C modulate the functional disconnection of weak inputs at polyinnervated neonatal neuromuscular synapses. J Neurosci Res.

[88] Garcia B, Lanuza (2019). Opposed actions of PKA isozymes (RI and RII) and PKC isoforms (cPKCβI and nPKCε) in neuromuscular developmental synapse elimination. Cells.

[89] Besalduch N, TomÃ s M, SantafÃ© MM et al (2010) Synaptic activity-related classical protein kinase C isoform localization in the adult rat neuromuscular synapse. J Comp Neurol 518:211–228. 10.1002/cne.22220 10.1002/cne.22220 19937712

[90] Obis T, Hurtado E, Nadal L (2015). The novel protein kinase C epsilon isoform modulates acetylcholine release in the rat neuromuscular junction. Mol Brain.

[91] Hurtado E, Cilleros V, Just L et al (2017) Synaptic activity and muscle contraction increases PDK1 and PKCβI phosphorylation in the presynaptic membrane of the neuromuscular junction. Front Mol Neurosci 10. 10.3389/fnmol.2017.00270 10.3389/fnmol.2017.00270 PMC5574929 28890686

[92] Thompson W, Kuffler DP, Jansen JKS (1979). The effect of prolonged, reversible block of nerve impulses on the elimination of polyneuronal innervation of new-born rat skeletal muscle fibers. Neuroscience.

[93] Brown MC, Hopkins WG, Keynes RJ (1982). Short- and long-term effects of paralysis on the motor innervation of two different neonatal mouse muscles. J Physiol.

[94] Duxson MJ (1982). The effect of postsynaptic block on development of the neuromuscular junction in postnatal rats. J Neurocytol.

[95] Callaway EM, van Essen DC (1989). Slowing of synapse elimination by α-bungarotoxin superfusion of the neonatal rabbit soleus muscle. Dev Biol.

[96] Simó A, Just-Borràs L, Cilleros-Mañé V et al (2018) BDNF-TrkB signaling coupled to nPKCε and cPKCβI modulate the phosphorylation of the exocytotic protein Munc18-1 during synaptic activity at the neuromuscular junction. Front Mol Neurosci 11. 10.3389/fnmol.2018.00207 10.3389/fnmol.2018.00207 PMC6007318 29946239

[97] Simó A, Cilleros-Mañé V, Just-Borràs L (2019). nPKCε mediates SNAP-25 phosphorylation of ser-187 in basal conditions and after synaptic activity at the neuromuscular junction. Mol Neurobiol.

[98] Leal K, Mochida S, Scheuer T, Catterall WA (2012). Fine-tuning synaptic plasticity by modulation of Ca _V_ 2.1 channels with Ca ^2+^ sensor proteins. Proc Natl Acad Sci.

[99] Lee A, Westenbroek RE, Haeseleer F (2002). Differential modulation of Cav2.1 channels by calmodulin and Ca2+-binding protein 1. Nat Neurosci.

[100] Garcia N, Lanuza MA, Besalduch N (2005). Localization of neuronal calcium sensor-1 at the adult and developing rat neuromuscular junction. J Neurosci Res.

[101] Incontro S, Díaz-Alonso J, Iafrati J (2018). Author correction: the CaMKII/NMDA receptor complex controls hippocampal synaptic transmission by kinase-dependent and independent mechanisms. Nat Commun.

[102] Bemben MA, Nguyen Q-A, Wang T (2015). Autism-associated mutation inhibits protein kinase C-mediated neuroligin-4X enhancement of excitatory synapses. Proc Natl Acad Sci.

[103] Lai ESK, Nakayama H, Miyazaki T et al (2021) An autism-associated neuroligin-3 mutation affects developmental synapse elimination in the cerebellum. Front Neural Circuits 15. 10.3389/fncir.2021.676891 10.3389/fncir.2021.676891 PMC8273702 34262438

[104] Bowling H, Klann E (2014). Shaping dendritic spines in autism spectrum disorder: mTORC1-dependent macroautophagy. Neuron.

